# A prospective comparison of total knee arthroplasty using ultra-congruent, condylar-stabilizing, and posterior-stabilized devices implanted with kinematic alignment: better 2-year outcomes with ultra-congruent

**DOI:** 10.1007/s00167-022-07206-w

**Published:** 2022-11-01

**Authors:** Brian J. Carlson, Brett K. Jones, David F. Scott

**Affiliations:** 1 Elson S. Floyd College of Medicine Washington State University, 412 E. Spokane Falls Blvd., Spokane, WA 99202 USA; 2 Spokane Joint Replacement Center, Orthopaedic Specialty Clinic of Spokane, Elson S. Floyd College of Medicine Washington State University, 785 East Holland Ave., Spokane, WA 99218 USA

**Keywords:** Total knee arthroplasty, PCL-sacrificing total knee arthroplasty, Ultra-congruent device, Posterior stabilized device, Condylar stabilizing device, Kinematic alignment

## Abstract

**Purpose:**

This study compared the 5-year results of posterior cruciate ligament (PCL)-sacrificing total knee arthroplasty (TKA) with either a post and cam posterior-stabilized (PS) device, a dished, congruent condylar-stabilizing (CS) device, or a deep-dished ultra-congruent (UC) device. The hypothesis was that the clinical and radiographic outcomes would be equivalent. CS and PS participants were part of a prospective, randomized trial, and UC participants were part of a separate prospective, non-randomized protocol that was otherwise identical. A kinematic alignment surgical technique was utilized.

**Methods:**

Participants were assessed preoperatively, and postoperatively at 6 weeks, 6 months, and annually for 5 years by Knee Society Score (KSS), SF-36 v2, Lower Extremity Activity Scale (LEAS), and physical and radiographic evaluation. There were 116 CS/PS participants and 69 UC participants who participated in the study.

**Results:**

Tourniquet (*P* = .02) and operative (*P* = .01) times for the CS and UC groups were significantly shorter than the PS group. KSS Function scores were better for the UC group than the CS and PS groups at 6 months (*P* = .04) and 1 year (*P* = .03), and better in the UC group vs. CS at 2 years (*P* = .04). The KSS Pain-only score was also better in the UC compared to PS at 6 months (*P* = .04). There were no significant differences for the KSS Pain/Motion scores, flexion, SF-36, and LEAS scores at any time.

**Conclusion:**

These data confirm the hypothesis that there are no clinically meaningful significant differences in outcomes between the three groups at a 5-year minimum follow-up, though there is a trend toward less pain and better function at earlier visits in the UC group.

**Level of evidence:**

II.

## Introduction

The traditional post and cam-style posterior-stabilized (PS) total knee device has been widely used, since it first ushered in the era of modern total knee arthroplasty and remains commonly used today. The ultra-congruent design was introduced as an alternative option for PCL substitution [26]. However, the relative merits of the PS device versus alternative devices such as a congruent condylar-stabilizing (CS) or the ultra-congruent (UC) device have not been fully elucidated [12].

The UC design has potential advantages, namely that the congruency of the polyethylene insert may distribute surface loads better, thus potentially reducing wear and providing greater knee stability [19]. The UC device does not require additional bone preparation necessary with the PS design, which preserves femoral bone and also likely reduces operative time and possibly blood loss [28], as well as a lower incidence of postoperative mechanical sensations, such as clicking, clunking, and popping [26]. Additionally, the anteroposterior constraint of the UC device is present through the full flexion arc, in contrast to the PS insert that does not engage until later in flexion. However, it is unknown whether the differences in articular geometry of these devices, with the potential for varying degrees of stability, may influence outcomes.

**Fig. 1:**
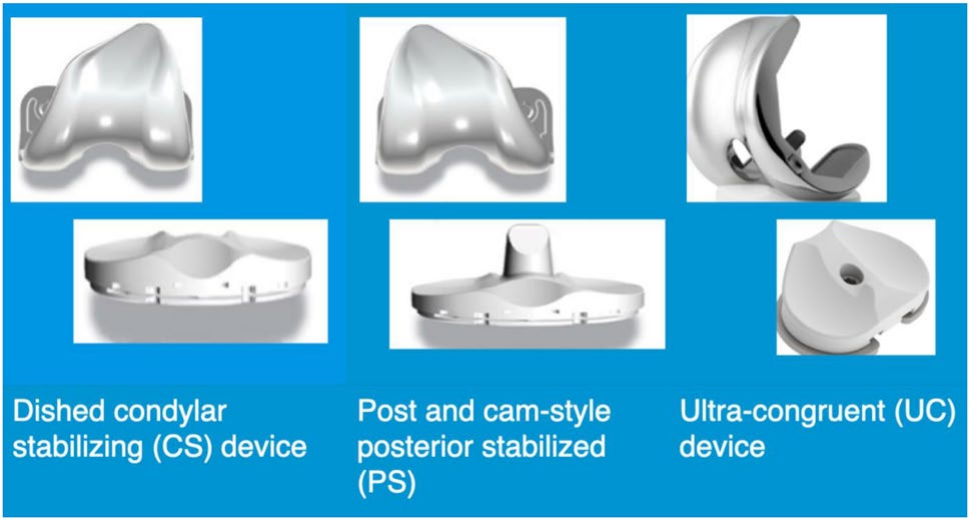
Implant designs for condylar stabilized, posterior stabilized, and ultra-congruent

The purpose of this prospective study was to compare the 5-year clinical outcomes and radiographic results of patients undergoing PCL-sacrificing total knee arthroplasty (TKA) receiving either a dished, congruent CS device, a PS device, or a UC device (Fig. 1). The hypothesis was that the clinical and radiographic outcomes achieved with these devices would be equivalent, with a trend favoring the UC.

## Materials and methods

A consecutive series of patients underwent primary TKA using one of three implant devices. The implants studied were 1) a dished, congruent CS device (Stryker Triathlon® CS), 2) a PS device (Stryker Triathlon® PS, Mahwah NJ), and an ultra-congruent device (Apex Knee, Corin Ltd., Raynham, MA). The participants who received the CS and PS devices were part of a prospective, randomized trial [26, 28], and the participants who received the ultra-congruent UC device were part of a separate prospective, non-randomized protocol that was otherwise identical. All other protocol variables were held constant. Participant inclusion and exclusion criteria are included in Table 1. The Stryker Triathlon CS device has an insert with an anterior jump height of 4 mm, while the Apex Knee is a true deep-dish design with an anterior jump height of > 10 mm. The femoral components of all three implants have a single-radius design.

**Table 1:** Inclusion and exclusion criteria

Inclusion criteria:
1. Willingness to understand and sign the informed consent
2. Ability to comply with study requirements including stress radiographs and evaluations
3. Male and non-pregnant female patients ages 21–80 years of age at the time of surgery
4. Patients who have undergone a primary TKA, are ≥ 1 year postoperative, without any clinical or radiographic evidence of failure
5. Patients enrolled in concurrent prospective randomized trials at study site
Exclusion criteria:
1. History of inflammatory arthritis
2. Declined to provide consent
3. Morbidly obese (BMI > 40 kg/M^2^)
4. History of total or unicompartmental reconstruction of the affected joint
5. High tibial osteotomy or femoral osteotomy
6. Neuromuscular or neurosensory deficiency
7. Systemic or metabolic disorder leading to progressive bone deterioration
8. Immunologically compromised
9. Arthrodesis of the affected joint
10. Active or suspected infection in or about the knee joint

All participants were operated on by the same surgeon, the senior author, and their TKAs were performed using identical kinematic alignment surgical technique, medial parapatellar arthrotomy, measured resection, intramedullary femoral, extramedullary tibial alignment, and posterior referencing without computer-assisted orthopaedic surgery (CAOS). In all cases, a tourniquet was inflated to 300 mm Hg prior to skin incision and deflated prior to closure once the final components were cemented. All posterior cruciate ligaments (PCLs) were sacrificed, and patellae resurfaced. All devices were cemented, using a surface-cementing technique on the tibia and carbon-dioxide lavage [6] with medium-viscosity Simplex P cement (Stryker, Kalamazoo, MI, USA). The femur was positioned in neutral rotation with respect to the posterior condyles, adjusting for cartilage loss and, when present, bone loss. The posterior tibial slope and proximal tibial varus was individually matched, accounting for cartilage and bone loss, attempting to recreate each patient’s unique pre-arthritic joint line. Ligament releases beyond the creation of a medial soft-tissue sleeve during the initial exposure were performed only in the presence of a significant valgus–flexion deformity. An unrestricted KA technique was utilized with manual instruments without boundaries for deformity, and caliper verification was utilized. The intramedullary femoral guide was adjustable to allow matching the distal femoral valgus angle, adjusting for cartilage loss.

Participants were assessed preoperatively, and at 6 weeks, 6 months, 1 year, and annually, until a follow-up of 5 years was reached. Clinical assessments included Knee Society Score, SF-36 v2, Lower Extremity Activity Scale (LEAS), radiographic evaluation, surgical data (i.e., intraoperative blood loss, tourniquet time, and operative time), complications, and adverse events. The radiographic analysis included long-limb standing views obtained preoperatively, and at 6 weeks and 1 year postoperative. We measured the coronal plane knee axes using a long-axis goniometer with 1º measurement increments. We measured active range of motion of the knee with the patient in a sitting position, utilizing a large goniometer with 1º increments.

The first CS/PS participant was consented on July 28, 2008, and enrollment of the last participant was completed in September 2010*.* The first UC participant was enrolled in August 2010 and the last in October 2013.

### Statistical analysis

Clinical trial management/electronic data capture/statistical analysis research software (Ortho Research Master®, Spokane Joint Replacement Center, Spokane, WA, USA) was utilized to compare the groups using Chi-square testing, Student’s *t* test, and Analysis of Variance (ANOVA) with Tukey post hoc with a significance level of 0.05.

### Ethics

All patients undergoing elective primary TKA were eligible for study recruitment. The Western Institutional Review Board granted approval (#20,100,725) for this study and all participants provided written informed consent. Strict adherence to inclusion/exclusion criteria was maintained, and good clinical practice and all regulatory mandates were followed. This study was registered and maintained on Clinicaltrials.gov, NCT Identifier # NCT01367938.

## Results

The mean follow-up period was 64 months (range, 60–66 months). The mean values of the age at surgery, BMI, and gender were compared between the implant groups, and no significant differences were found between groups (Table 2).

**Table 2:** Demographic data

Variable	CS	PS	UC	*P*
Men/women (*n*)	21/37	29/29	33/36	.54
Mean age (years)	61.9	66.0	64.4	.06
Men/women	63.8/60.9	65.7/66.4	63.5/65.4	.62
Mean BMI^a^ men/women	31.9/33.5	31.5/32.7	32.3/31.7	.77

^a^Body mass index: kg/M^2^

There were 116 CS/PS participants screened and enrolled by September 2010, 14 participants were lost to follow-up during the study, and 109 active participants completed the study. There were 69 UC participants enrolled, 21 UC participants either withdrew consent or were lost to follow-up during the study, and 48 UC participants completed the study. There were no infections requiring surgery. There have been no adverse events or serious adverse events attributed solely to the CS and PS devices. Two CS/PS participants required additional surgery (one patella fracture 6 months postoperatively and one loosening of the tibial baseplate after an automobile accident 1 year postoperatively). Excluding these two reoperations, CS/PS implant survivorship was 100%. One UC participant underwent reoperation for a loosened polyethylene insert locking bolt, requiring surgical intervention 3 years postoperatively.

Intraoperative data are shown in Table 3. Tourniquet times for the CS and UC groups were significantly shorter than the PS group (*P* = 0.02). The operative time for the CS and UC groups was also significantly lower than the PS group (*P* = 0.01) (Table 3). Knee Society Function scores were better for the UC group than the CS and PS groups at 6 months (*P* 0.04) and 1 year (*P* = 0.03). There was also a difference in the KSS Function score at 2 years, favoring the UC vs. the PS group (Table 4). There was a statistical difference in the KSS pain-only score as well, also favoring the UC group at 6 months. There were no differences in the Knee Society Pain/Motion scores. There were no statistically significant differences among the three groups preoperatively, at 6 weeks, and at the 2-, 3-, 4-, and 5-year assessments in the scores. There were no significant differences in the SF-36 scores at all time points. The LEAS assessment found only one statistically significant result for UC vs. CS at 5 years postoperatively (Table 5). Radiographic assessment found only one statistically significant difference for tibiofemoral alignment for UC vs. PS at the preoperative assessment (*P* = 0.04) (Table 6). Range of motion measurement found only one significant difference for UC vs. CS extension at the 4-year postoperative assessment (*P* = 0.03) and there were no significant differences in flexion among devices at any follow-up time point (Table 7).

**Table 3:** Tourniquet and operative times

	CS	PS	UC	*P*
Mean tourniquet time (min.)	33.4/4.7	36.5/5.5	32.3/6.9	.02
Mean operative time (min.)	49.6/5.4	54.7/7.7	49.2/10.4	.01

**Table 4:** Knee society scores (mean)

Implant group	Function		Pain/motion	
	*n* Participants	Score/std. dev	*n* Participants	Score/std. dev
CS				
Preoperative	58	52.07/13.89	55	47.02/13.38
6 weeks	58	58.88/17/47	55	72.96/15.46
6 months	55	73.36/19.13	53	89.57/12.38
1 year	48	78.54/20/08	45	90.31/10.74
2 years	35	80.71/19.03	34	94.06/10.89
3 years	36	90.28/12.36	36	97.61/3.96
4 years	30	84.33/23.22	30	97.43/3.23
5 years	30	86.50/28.01	30	96.90/8.41
PS				
Preoperative	58	53.02/13.67	54	48.87/10.45
6 weeks	58	56.47/18.21	54	75.65/15.09
6 months	54	76.39/19.34	50	88.20/13.63
1 year	54	80.0/18.53	51	91.35/12.71
2 years	38	85.13/18.98	37	95.19/8.08
3 years	37	90.27/14.09	35	97.49/3.98
4 years	37	88.24/20.15	37	95.76/9.96
5 years	35	86.57/17.31	33	96.21/6.94
UC				
Preoperative	69	58.19/18.51	69	52.26/12.38
6 weeks	69	63.04/22.59	69	77.03/18.96
6 months	65	87.69/18.79	65	91.97/11.15
1 year	56	91.88/13.47	56	95.07/10.07
2 years	47	91.06/14.44	47	95.30/6.58
3 years	45	90.56/16.66	45	95.91/7.15
4 years	34	90.74/19.93	34	96.79/6.25
5 years	40	87.63/21.36	41	97.73/3.15

**Table 5:** LEAS, SF-35, and SF-36 PCS scores (mean)

Implant group	LEAS		SF-36 MCS		SF-36 PCS	
	*n* Participants	Score/std. dev	*n* Participants	Score/std. dev	*n* Participants	Score/std. dev
CS						
Preoperative	58	8.05/2.61	56	50.76/11.33	56	31.49/7.36
6 weeks	58	8.14/2.38	57	49.20/10.56	57	36.67/8.33
6 months	55	10.49/2.84	54	53.94/9.13	54	45.12/9.42
1 year	48	10.56/2.89	47	52.08/9.13	47	45.38/8.92
2 years	35	10.74/2.94	35	53.50/9.35	35	44.40/8.91
3 years	36	9.69/2.68	36	51.09/11.14	36	43.15/10.31
4 years	30	9.47/2.21	30	53.97/9.21	30	44.54/9.64
5 years	30	8.97/2.55	29	52.20/10.50	29	43.10/12.04
PS						
Preoperative	58	7.88/2.48	58	51.70/11.15	58	32.85/7.47
6 weeks	58	7.91/2.29	57	50.08/10.32	57	35.90/7.82
6 months	54	10.72/2.98	54	52.35/10.65	54	45.37/8.79
1 year	54	10.87/3.11	54	53.48/11.69	54	45.06/10.95
2 years	38	10.26/3.21	37	53.00/9.72	37	45.29/10.32
3 years	37	10.57/3.30	37	51.47/11/24	37	47.03/8.96
4 years	37	9.70/2.82	37	53.65/9.48	37	43.19/11.13
5 years	35	10.09/3.51	35	52.31/9.93	35	43.66/9.88
UC						
Preoperative	68	8.78/2.69	69	49.20/12.52	69	32.50/7.49
6 weeks	69	8.45/2.89	69	48.93/11.39	69	34.83/7.62
6 months	65	10.75/3.06	65	53.28/9.81	65	44.13/9.37
1 year	56	10.75/2.99	56	53.53/8.89	56	44.50/9.64
2 years	47	10.72/3.09	47	55.07/7.13	47	44.72/9.44
3 years	45	10.36/2.97	45	50.98/9.65	45	54.25/11.02
4 years	34	10.35/2.75	34	54.26/9.50	34	43.86/10.40
5 years	41	10.98/3.31	41	55.13/9.78	41	44.82/10.47

**Table 6:** Radiographic results: anatomic tibiofemoral angle (mean)

Anatomic tibiofemoral angle°			
Implant group	*n* participants	Value/std. dev	*P*
CS			
Preoperative	58	2°/6°	.04^a^
6 weeks	55	6°/2°	.24
1 year	47	6°/2°	.41
PS			
Preoperative	58	1°/5°	.04
6 weeks	53	5°/3°	.24
1 year	48	5°/2°	.41
UC			
Preoperative	68	4°/6°	.04
6 weeks	69	6°/2°	.24
1 year	51	6°/2°	.41

^a^The only statistically significant difference is between the PS and UC groups at the preoperative visit

**Table 7:** Range of motion results (mean)

Implant group	*n* Participants	Flexion°/std. dev	Extension°/std. dev
CS			
Preoperative	58	114.17/13.48	4.93/5.97
6 weeks	58	109.50/17.39	4.76/6.03
6 months	55	123.00/11.72	1.62/3.42
1 year	48	124.17/11.49	1.06/2.76
2 years	35	125.46/9.68	0.57/2.65
3 years	36	127.53/13.85	0.28/1.16
4 years	30	126.10/8.71	^a^0.00/0.00
5 years	30	123.87/13.56	0.33/1.27
PS			
Preoperative	58	113.45/10.77	6.21/6.83
6 weeks	58	112.95/14.73	6.86/5.92
6 months	54	122.80/9.03	2.52/4.80
1 year	54	125.98/7.26	1.07/3.10
2 years	38	126.89/6.49	0.74/2.67
3 years	37	127.95/6.14	0.46/1.83
4 years	37	127.97/5.60	0.49/1.87
5 years	35	125.03/7.92	0.57/2.02
UC			
Preoperative	69	114.72/12.15	5.22/5.59
6 week	69	113.09/15.57	5.88/6.07
6 month	65	122.97/9.83	2.15/4.12
1 year	56	126.84/7.39	0.82/2.26
2 years	47	125.06/8.55	1.21/2.78
3 years	45	125.84/8.76	0.51/1.49
4 years	34	123.79/11.48	^a^1.21/2.35
5 years	41	125.73/8.31	0.39/1.24

^a^Statistically significant *P* = .03. The only statistically significant difference is between the CS and UC groups’ extension at the 4-year visit

## Discussion

The most important finding of this study was that the hypothesis was confirmed: the clinical and radiographic outcomes achieved with these devices would be equivalent at 5 years, with a trend favoring the UC group. The ROM, Knee Society Pain/Motion Score, LEAS, SF-36 scores, and radiographic outcomes are all statistically similar. However, the KSS Pain-only and the KSS Function scores favored the non-PS groups, primarily favoring the UC group. Additionally, the tourniquet and total operative times were longer for the PS group compared to the CS and UC groups.

There was only one device-related complication, in the UC group: one participant whose polyethylene insert locking bolt was found to be loose at the 3-year follow-up visit, requiring reoperation. This issue has been addressed with a modification in the instrumentation to include an anti-rotational handle to ensure full torque application during assembly of the locking bolt, without affecting tibial baseplate stability and cement fixation during the cement curing process.

Hofmann, et al. [8] were one of the first to report favorable results with the use of a UC insert. Other early studies reported favorable results with the use of a dished insert with PCL recession or sacrifice [25]. Studies have compared UC clinical results with cruciate-retaining devices, in some cases reporting no differences [31]. Other comparisons of CR and UC devices have reported advantages to the UC option. In a large non-randomized study, Berend et al. found a greater improvement in range of motion and KSS scores but a higher manipulation rate in the UC group compared to CR, as well as better survivorship for UC vs CR [2]. Peters, et al. [22] compared a UC to a CR bearing using a single femoral component in a retrospective review of 468 TKRs with a minimum 2-year follow-up, and found no differences in Knee Society scores, radiographic alignment, component loosening, and rate of manipulation. However, there were 21 CR revisions vs. 7 UC revisions (*P* = 0.03) and the UC group had significantly better survival at 5 years (97% vs. 88%, *P* = 0.01). Of the 21 CR revisions, 6 were for instability.

In a randomized study of 107 knees receiving either a fixed-bearing UC or PS device at 5-year follow-up, it was found that surgical time was 10 min shorter in the UC group (*P* < 0.001). Both groups demonstrated equally good knee function, quality of life, and high satisfaction. Patellofemoral problems were more frequent in the PS group (*P* = 0.025) [17]. In a report of the results from a large national registry, Dalton found that survivorship was better for a UC device versus a PS device: the cumulative revision at 18 years was 8.3% for UC, 9.2% for CR, and 8.9% for PS [3].

A 2016 prospective computer-aided navigation study of a UC device and a PS rotating platform device correlated passive kinematics to clinical outcomes [15]. They measured anterior/posterior translation, varus/valgus alignment, and femoral rotation during passive flexion. The UC knees had greater anterior translation in higher flexion (from 60° to 90º of flexion), but less abnormal external rotation than the PS group. Neither device was able to reproduce physiologic knee kinematics. There were no significant differences between implant groups for ROM and outcomes scores, supporting the UC device as an alternative to the PS device.

A report [24] of a sit-to-stand test with dynamic radiostereometric analysis comparing UC and PS designs found that there was significantly more anterior translation in the PS group versus UC between 0°and 30° of flexion, concluding that the UC design possessed greater AP stability. The PS implant surpassed the UC in AP stability after greater than 45º of flexion. This is very interesting, because the majority of activities of daily living require knee stability in extension to mid-flexion, not 90º of flexion [4, 11, 30, 32, 33], yet most of the reports in the literature focus on stability measurements limited to 90º of flexion, ignoring mid-flexion stability.

Illustrating this point that stability in mid-flexion is more relevant clinically than stability in 90º of flexion, Jang performed a study [10] in 45 bilateral TKA patients with a UC on one side and a PS on the other, and found that, despite a difference in static laxity at 90º of flexion at 2-year follow-up, there were no differences in dynamic AP stability evaluated using a one-leg standing lateral fluoroscopic imaging throughout the range of motion at 30°, 60°, and 90° knee flexion. There was also no difference in ROM, or WOMAC or KSS scores.

In another dynamic fluoroscopy evaluation, Khasian found that patients with a UC device experienced femoral rollback of the lateral condyle and a normal pattern of axial rotation with flexion, although lower in magnitude than the normal knee. Most importantly, these authors reported minimal dynamic mid-flexion laxity with this UC device [13].

The kinematic alignment approach used in this study may affect the results in a manner that might not be directly comparable to a more traditional mechanical alignment and/or gap balancing surgical approach. It is possible that the UC knee design is better suited for use with kinematic alignment, which preserves the native knee anatomy of each individual patient more closely than other alignment techniques. Perhaps, the PS and CS knee would perform better with a different surgical technique such as mechanical alignment. It is also theoretically possible that the results seen with this specific implant may not be translated to other brands of UC, CS, and/or PS knee systems. To the best of our knowledge, this is the first study reporting the results with a UC device implanted with kinematic alignment.

The main limitation of this study is that we did not include the Forgotten Joint Score, which may help discriminate between these implant designs, since kinematic alignment in and of itself provides a high ceiling effect for other scoring schemes such as the Knee Society Score. Another limitation is that the UC group was not part of the randomized CS vs PS group, but instead it was a consecutive group of subjects enrolled after the CS and PS subjects. This could theoretically influence the data in that there might be some subtle differences in various factors such as rehabilitation, pain management, etc.; however, the surgical technique employed was identical. Another limitation is that our enrollment numbers are modest in size, especially at the longer follow-up periods, and that it is possible that with a greater number of patients enrolled statistically significant differences in outcomes might be detected.

The PS knee design has several negative attributes not shared with other designs, including a greater incidence of the presence of mechanical sensations [14, 26], greater blood loss [18, 28], greater femoral bone loss due to preparation of the notch with the box cut, and higher tourniquet times [21, 28]. There are other complications unique to the PS design, including femoral condylar fracture [1], polyethylene wear [23] and fatigue fracture of the post [7], dislocation [16], and patellar clunk syndrome [9, 29]. Additionally, in comparison with a different, more constrained implant design, a medial-stabilized implant, the PS implant has produced inferior clinical results with kinematic alignment [27]. Another study utilizing this same more constrained, medial-stabilized knee with kinematic alignment, reported superior Forgotten Joint Scores versus a CR implant [5]. Currently, the continued use of the PS knee in primary knee arthroplasties has been questioned [20].

## Conclusion

This prospective consecutive series of PCL-sacrificing TKA implanted with kinematic alignment failed to find major differences in clinical outcomes between UC, CS, and PS knee groups through 5 years of follow-up, though there was a trend favoring the UC group between 6 months and 2 years. These results provide support for clinical use of the UC device as an alternative to the PS and CS devices.

## Data Availability

The datasets used and analyzed during the current study are available from the corresponding author on reasonable request.
